# Mechanism of FGF receptor dimerization and activation

**DOI:** 10.1038/ncomms10262

**Published:** 2016-01-04

**Authors:** Sarvenaz Sarabipour, Kalina Hristova

**Affiliations:** 1 Department of Materials Science and Engineering, Johns Hopkins University, Baltimore, Maryland 21218, USA

## Abstract

Fibroblast growth factors (fgfs) are widely believed to activate their receptors by mediating receptor dimerization. Here we show, however, that the FGF receptors form dimers in the absence of ligand, and that these unliganded dimers are phosphorylated. We further show that ligand binding triggers structural changes in the FGFR dimers, which increase FGFR phosphorylation. The observed effects due to the ligands fgf1 and fgf2 are very different. The fgf2-bound dimer structure ensures the smallest separation between the transmembrane (TM) domains and the highest possible phosphorylation, a conclusion that is supported by a strong correlation between TM helix separation in the dimer and kinase phosphorylation. The pathogenic A391E mutation in FGFR3 TM domain emulates the action of fgf2, trapping the FGFR3 dimer in its most active state. This study establishes the existence of multiple active ligand-bound states, and uncovers a novel molecular mechanism through which FGFR-linked pathologies can arise.

## Abstract (web summary)

Different fibroblast growth factor (fgf) ligands elicit specific biological responses, but the mechanism directing this specificity is unknown. Here the authors show that fgf1 and fgf2 impose specific reorganizations on the fgf receptor dimer trans-membrane helices that result in distinct level of receptor activation.

## Graphical abstract

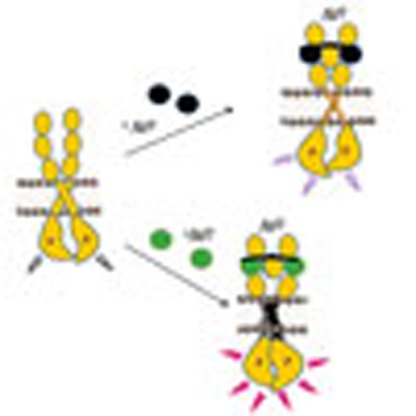

The fibroblast growth factor receptor (FGFR) family includes four receptors that bind 18 ligands called fibroblast growth factors, using heparin as a co-factor^1^,^2^,^3^,^4^. These receptors play important roles in all cell types, but are best known for the critical role that they play in the development of the skeletal system^5^. Many pathogenic mutations of FGFR genes are linked to skeletal, cranial and other developmental abnormalities in humans^6^,^7^. Furthermore, FGFR overexpression and mutations have been reported in a variety of cancers^8^,^9^,^10^,^11^,^12^,^13^,^14^,^15^.

FGF receptors are single-pass membrane proteins, with N-terminal extracellular (EC) domains consisting of three immunoglobulin-like subdomains (D1, D2 and D3), a transmembrane (TM) domain consisting of a single α-helix, and an intracellular (IC) region encompassing a tyrosine kinase domain^16^,^17^,^18^. FGFRs transduce biochemical signals via lateral dimerization in the plasma membrane. Receptor dimerization is necessary for activation, as it brings the two tyrosine kinase domains into close proximity, allowing them to cross-phosphorylate each other on tyrosines in their activation loops^2^,^19^. This activates the kinases, which then bind adaptor proteins and phosphorylate cytoplasmic substrates, triggering downstream signalling cascades that control cell growth and differentiation^20^,^21^,^22^.

High-resolution crystal structures of isolated FGFR EC domains in the presence of different fgfs have provided detailed views of ligand–receptor and receptor–receptor interactions in the EC portion, as well as the role of the co-factor heparin^23^,^24^,^25^,^26^,^27^. However, there is little mechanistic understanding of how conformational changes are transmitted from the EC domains through the TM domains to the kinase domains, in response to ligand binding. Different fgf ligands can elicit distinctly different biological responses^28^, but the mechanism behind the specificity is unknown. To gain insight into these issues, here we study the dimerization of FGFR1, FGFR2 and FGFR3, as well as the response of these receptors to the ligands fgf1 and fgf2. Our results show that ligand binding to unliganded FGFR dimers triggers a switch to ligand-specific configurations of the TM helices, which in turn increase receptor phosphorylation. We further show that a pathogenic FGFR mutant causes unregulated ligand-independent signalling by mimicking the most active ligand-bound configuration.

## Results

### Full-length FGF receptors dimerize in the absence of ligand

FGF receptors have been proposed to form dimers in the absence of ligand^29^,^30^,^31^,^32^. However, the dimerization propensities of unliganded FGF receptors have not been measured directly or quantified. We used a Förster resonance energy transfer (FRET)-based technique, ‘Quantitative Imaging FRET' or QI-FRET^33^, to quantify the dimerization of full length FGFR1, FGFR2 and FGFR3 in plasma membrane-derived vesicles obtained directly from mammalian cells. While they lack cell cytoplasm, the vesicles are composed of lipid and proteins that are native to the plasma membrane^34^. Two-dimensional receptor concentrations are readily measurable in these vesicles, allowing us to quantify the physical interactions between membrane receptors^34^,^35^,^36^.

**Figure 1:**
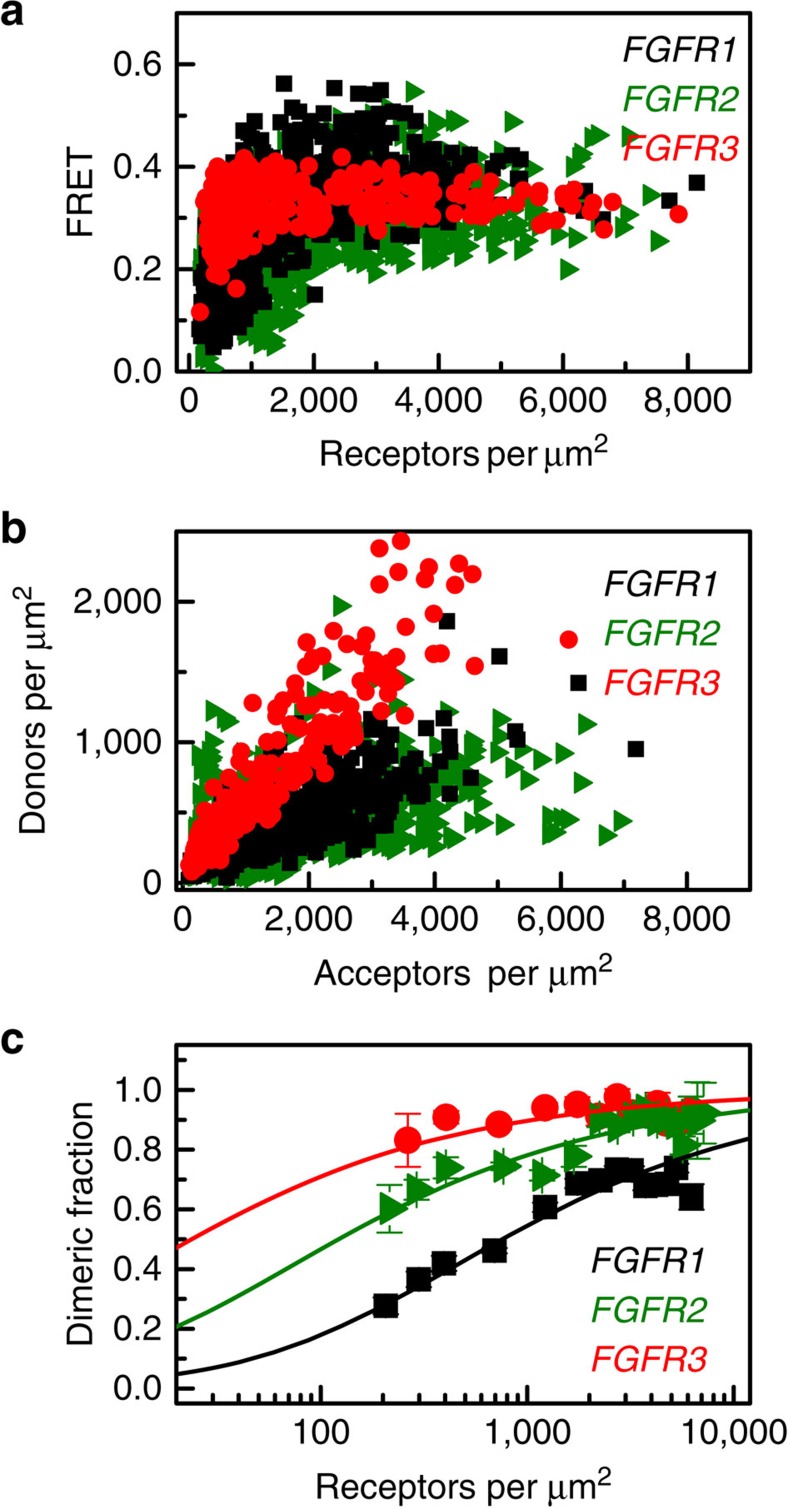
FGF receptor dimerization in the absence of ligand. (**a**) Measured FRET in plasma membrane-derived vesicles, as a function of receptor concentration, for FGFR1 (black), FGFR2 (olive) and FGFR3 (red). Every data point represents a single vesicle. (**b**) The donor concentration is plotted as a function of the acceptor concentration, for each vesicle. (**c**) Dimeric fraction as a function of total receptor concentrations. The experimentally determined dimeric fractions are binned and are shown with the symbols, along with the standard errors. Each bin contains between 5 and 50 experimental points. The solid lines are the dimerization curves, plotted for the optimized dimerization parameters in Table 1.

The monomeric fluorescent proteins YFP or mCherry are a FRET pair suitable for QI-FRET^37^. Single YFP or mCherry genes were fused to the C termini of the full-length FGFR1, FGFR2 and FGFR3 genes via a sequence encoding for a flexible GGS linker (Supplementary Fig. 1). For each of the FGF receptors, Chinese Hamster Ovary (CHO) cells were co-transfected with two plasmids, one encoding FGFR-YFP and one encoding FGFR-mCherry. After the receptors were expressed and trafficked to the plasma membrane, the cells were vesiculated using gentle osmotic stress method^38^,^39^. The vesicles were collected and imaged, in the absence of ligand, using laser scanning confocal microscopy^35^,^36^,^40^ (Supplementary Fig. 3). For each vesicle, we used the images captured in the donor, acceptor and FRET channels to determine the donor concentration, the acceptor concentration, the total receptor concentration, the FRET efficiency and the receptor dimeric fraction. As shown in Fig. 1a,b, 800–1200 individual plasma membrane-derived vesicles were analysed in 3–5 independent experiments for each FGF receptor. The single-vesicle data were combined to yield dimerization curves for the receptors, as described in the Methods section under ‘QI-FRET Data Analysis'.

The dimeric receptor fraction as a function of receptor concentration is shown in Fig. 1c. From this concentration dependence we obtained, by fitting, the two-dimensional dissociation constant *K*_diss_ and the structural parameter ‘intrinsic FRET', 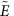 (refs 33, 41; Table 1). Intrinsic FRET does not depend on the dimerization propensities, and is directly related to the distance between the fluorescent proteins. As discussed below, measurements of intrinsic FRET allow us to capture structural changes that occur on the cytoplasmic side of the receptor on ligand binding to the extracellular domains.

The values of the two-dimensional dissociation constants, *K*_diss_ are 710, 111 and 24 μm^−2^ for FGFR1, FGFR2 and FGFR3, respectively, corresponding to dimerization free energies of −4.3±0.1, −5.4±0.1 and −6.3±0.1 kcal mol^−1^ (see equation (10) and Table 1; uncertainties are standard errors). The intrinsic FRET values for the unliganded FGFR1, FGFR2 and FGFR3 dimers are 0.66, 0.43 and 0.55, respectively (Table 1).

To evaluate the biological significance of the measured unliganded dimerization of the FGFRs, we note that physiological FGFR expression levels can be as high as ∼80 000 receptors per cell, corresponding to ∼80–100 receptors per μm^2^ (ref. 42). The experimental dimerization curves that we measured for the three receptors, shown in Fig. 1c, predict substantial dimer populations, at least for FGFR2 (∼20%) and FGFR3 (∼50%), at receptor concentrations as low as 10 receptors per μm^2^. Furthermore, we see a substantial increase in dimeric fraction with concentration, consistent with reports that FGFR overexpression is linked to cancer^10^,^11^,^12^,^13^,^14^,^15^. Thus, unliganded FGFR dimerization is important in physiological context.

### Contributions of FGFR domains to unliganded dimerization

To determine the contribution of individual domains to the energetics of dimerization of the unliganded FGF receptors, we created two truncated versions of each receptor. In one truncated version (EC+TM), the intracellular domains were removed and the fluorescent proteins were attached to the cytoplasmic end of the TM domains via flexible, 15 residue (GGS)_5_ linkers^43^ (Supplementary Fig. 1). In the second truncated version (TM), both the IC and EC domains were removed, so that these constructs only contained the TM domains attached to the fluorescent proteins via the same flexible (GGS)_5_ linkers (Supplementary Fig. 1). In earlier work, we showed that the attachment of the fluorescent protein to the TM domain via this linker does not have an effect on dimerization^36^.

**Figure 2:**
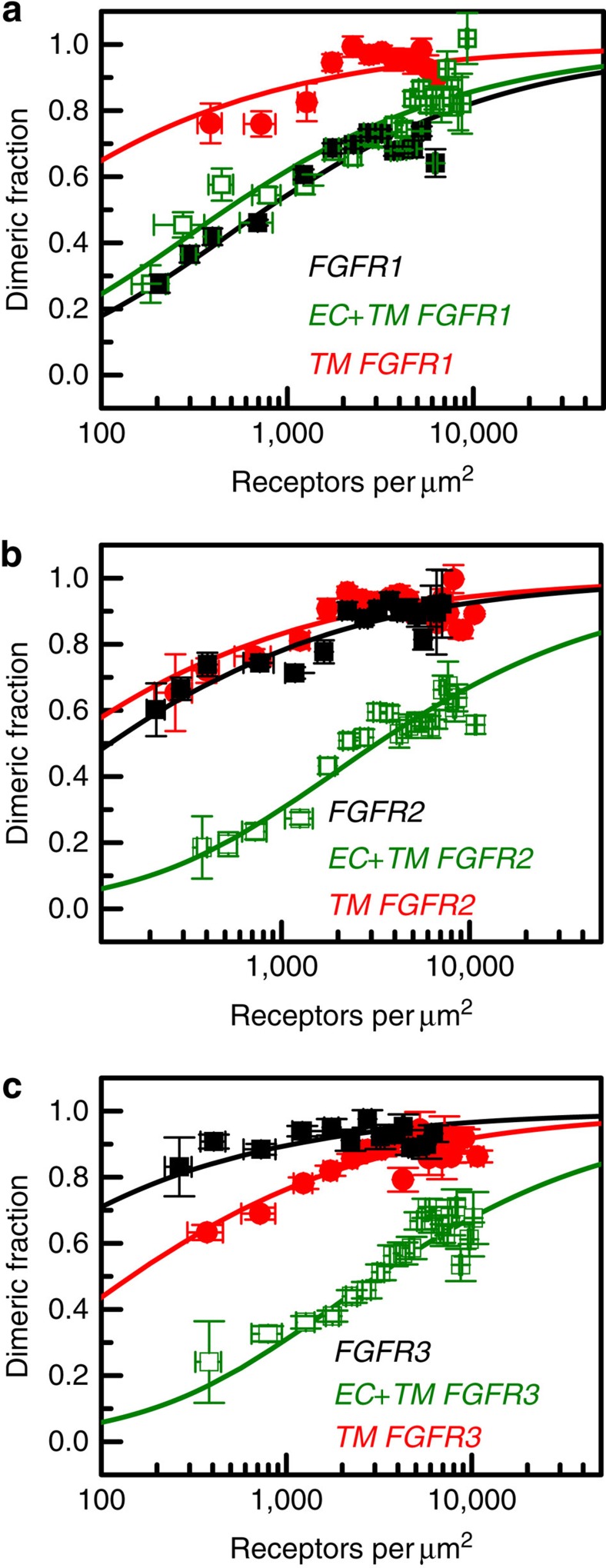
FGFR domain contributions to unliganded dimerization. Dimerization curves are shown for the full-length receptors (black), for truncated receptors that lack the IC domain and thus contain only the EC and TM domains (olive), and for the TM domains only (red). (**a**) FGFR1. (**b**) FGFR2. (**c**) FGFR3. Data for EC+TM FGFR3 and TM FGFR3 are from ref. 36. The measured dimeric fractions are binned and are shown with the symbols, along with the standard errors. Each bin contains between 5 and 50 experimental points. The solid lines are the best fits of a monomer–dimer equilibrium model to the single-vesicle data. These data demonstrate that the TM domains have a strong propensity for dimerization. The EC domains, on the other hand, inhibit dimerization. The contribution of the IC domains is favourable, but it varies from zero to −3 kcal mol^−1^ for the three receptors.

The dimerization of the truncated constructs in plasma membrane-derived vesicles was characterized by QI-FRET as above. The dimerization curves for the truncated receptors are shown in Fig. 2, along with the results for the full-length receptors for comparison. The dimerization constants, free energies of dimerization and intrinsic FRET values for all the variants are shown in Table 1. These results reveal several important aspects of FGFR unliganded dimerization. First, they show that the TM domains alone have a strong propensity for dimerization, with dimerization free energies between −5.2 and −6.0 kcal mol^−1^. Second, they demonstrate that FGFRs that lack IC domains form dimers. It has been proposed previously that the IC domain is required for FGFR dimerization in the absence of ligand^44^,^45^. However, our results directly show that the IC domain is not necessary for FGFR dimerization. Third, the differences in stability of the two types of truncated receptors (EC+TM and TM) suggest that the contribution of the EC domains to unliganded FGFR dimerization is destabilizing for all three receptors, by 1.4–2.3 kcal mol^−1^.

Last, by comparing the stabilities of the full-length receptors to the truncated receptors without the IC domains, we obtained directly, and for the first time, the thermodynamic contribution of the IC domain to FGFR unliganded dimerization. Surprisingly, there are large differences in the IC domain contribution among the three receptors. While the contributions of FGFR2 and FGFR3 IC domains are stabilizing by −2.0 and −2.9 kcal mol^−1^, respectively, the contribution of FGFR1 IC domain is practically zero, suggesting that either FGFR1 IC domain does not engage in contacts that stabilize the full-length FGFR1 dimer, or that stabilizing contacts are balanced by repulsive ones.

### Structural changes in FGFR dimers on fgf1 and fgf2 binding

In the experiments described above, we determined the intrinsic FRET for all the studied dimers in the absence of ligand (Table 1). The intrinsic FRET is a structural parameter that is directly related to the distance between the fluorescent proteins, and that does not depend on the dimerization propensities. By comparing the intrinsic FRET in the presence and absence of ligand, here we investigated structural changes that occur on the cytoplasmic side of the receptor on ligand binding to the extracellular domains.

The interaction interface of the TM domains has been proposed to play a role in FGFR activation^46^,^47^, and we therefore looked at the effect of ligand on the truncated EC+TM FGFR constructs, in which the fluorescent proteins were attached to the TM domains via flexible, 15-residue (GGS)_5_ linkers. The intrinsic FRET values for the unliganded EC+TM FGFR1, FGFR2 and FGFR3 dimers were 0.50, 0.57 and 0.52, respectively (Table 1). Assuming random orientation of the fluorophores (justified because they were attached via flexible 15 amino acid long linkers^43^), we calculated the effective distances between the fluorescent proteins in the unliganded EC+TM dimers (Table 1).

Since the fluorescent proteins were attached directly to the TM domains via the flexible linker, we could directly monitor changes in the structure of the TM domains in the receptor dimers in response to ligand binding. Experiments were performed at high, saturating ligand concentration (5 μg ml^−1^) such that ligand concentration exceeded the ligand–receptor dissociation constants and the total FGFR concentration by at least two orders of magnitude as shown previously^48^,^49^,^50^. This ensured that all receptors were in the ligand-bound dimeric state^48^,^49^,^50^. In the case of 100% dimeric receptors, the FRET signal does not depend on receptor concentration (Supplementary Fig. 5). Instead, it depends only on the intrinsic FRET and on the acceptor fraction, which is measured in each vesicle (equation (11)).

**Figure 3:**
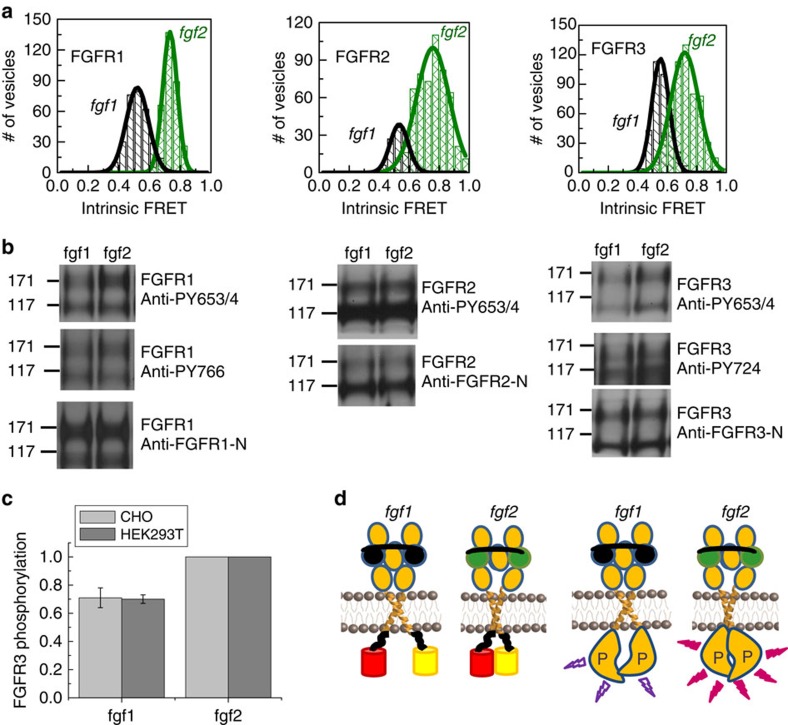
Conformational changes and activation of the FGF receptors. (**a**) Histograms of single-vesicle intrinsic FRET values, measured for the three FGF EC+TM receptor constructs in the presence of saturating fgf1 (black) or fgf2 (olive) concentrations. Intrinsic FRET is a measure of the separation between the fluorescent proteins in the dimer. Two different intrinsic FRET values were measured for fgf1 and fgf2. Therefore, the binding of these two ligands to the extracellular domains leads to different separation of the fluorescent proteins on the cytoplasmic side of the membrane (Table 2). (**b**) Western blots, reporting on the phosphorylation of the full-length receptors in the presence of saturating concentrations of fgf1 and fgf2 (5 μg ml^−1^). Expression of the receptors was probed with antibodies to the extracellular domains of the three receptors. Phosphorylation was assayed using antibodies against phosphorylated tyrosines in the activation loop of the three kinases (anti-phospho-Y653/4) or other phosphorylated tyrosine residues. Two bands are observed for all receptors. Only the top bands, corresponding to the fully glycosylated mature receptors that reside primarily in the plasma membrane, were considered in our analysis. There is a difference between the phosphorylation in response to fgf1 and fgf2 for FGFR1 and FGFR3, but not for FGFR2 (see text). (**c**) Relative FGFR3 phosphorylation in response to fgf1 and fgf2 is quantified and compared using a *t*-test. Five independent experiments were performed in two cell lines, CHO and HEK 293T. Phosphorylation was calculated by dividing the intensities of the anti-phospho-Y bands to the intensities of the anti-receptor bands, and scaled to the fgf2 case. The difference in FGFR3 phosphorylation in response to fgf1 and fgf2 is highly statistically significant (*P*<0.01). (**d**) Graphic representation of the findings that the fgf1- and fgf2-bound states are structurally and functionally distinct. Left: graphic (not to scale) representation of the finding that the average distance between the fluorescent proteins is larger when fgf1 is bound, as compared with the fgf2-bound case. Right: graphic representation of the finding that phosphorylation is higher when fgf2 is bound. The representation of the kinase domains is a cartoon, not based on structural data.

Histograms of intrinsic FRET values, measured in single vesicles for each receptor–ligand pair, are shown in Fig. 3a, and means and standard errors are given in Table 2. We see that intrinsic FRET depends only on the type of ligand, and not on the receptor identity. For all three receptors, FGFR1, FGFR2 and FGFR3, intrinsic FRET in the fgf1-bound dimer state was ∼0.55, while the intrinsic FRET value in the fgf2-bound dimer state was ∼0.73 (Table 2). These differences are statistically significant, *P*<0.01. The distances between the fluorescent proteins, calculated under the assumption of random fluorophore orientation using equation (12), are shown in Table 2. Note that these are the effective average distances as the linkers have been shown to behave as random coils and explore a variety of configurations^43^. In the presence of fgf1, the effective distance between the fluorescent proteins is 51.6±0.4 Å, which is similar to the unliganded case (*P*>0.1, Table 1). In the presence of fgf2, the effective distance between the fluorescent proteins is 44.9±0.6 Å, significantly smaller than in the fgf1-bound dimer (*P*<0.01). These measured differences in intrinsic FRET reflect differences in the separation of the C termini of the TM domains in the two ligand-bound states, diagrammed in Fig. 3d.

### Changes in FGFR phosphorylation on fgf1 or fgf2 binding

To investigate the biological significance of the two different ligand-bound states that we observed in the QI-FRET experiments, we compared the phosphorylation of full-length FGFR1, FGFR2 and FGFR3 at saturating fgf1 or fgf2 concentrations (5 μg ml^−1^) using western blotting. For detection of activated (phosphorylated) receptors we used anti-phospho-Tyr antibodies that are specific for phosphorylated tyrosines in the activation loop of the three receptors (anti-phospho-Y653/4) or other intracellular tyrosines. Typical western blot results are shown in Fig. 3b and Supplementary Fig. 6. Only the top bands, corresponding to the mature fully glycosylated receptors, were quantified. Comparing liganded FGFR1 and FGFR3, we consistently observe 20–40% higher phosphorylation in the presence of fgf2 than fgf1 in CHO cells. To test for statistical significance of this observation using a Student's *t*-test, we performed five independent FGFR3 experiments in two different cell lines, CHO and HEK 293T. In both cell lines, activation by saturating fgf2 is 40% higher than for fgf1 (Fig. 3c). The calculated *P* value is <0.01, showing that the difference is highly statistically significant. The western blot results therefore support the finding of two distinct ligand-bound active FGFR1 and FGFR3 states, and demonstrate that these different structural states correlate with biological activity.

We used the same method to measure phosphorylation in the absence of ligand. Supplementary Figure 6 shows typical western blot results in the absence of ligand, along with the fgf1 and fgf2 results. For FGFR1 and FGFR3, the phosphorylation in the absence of ligand was 35–65% of the phosphorylation in the presence of fgf1. Thus, the unliganded FGFR1 and FGFR3 dimers exhibit significant phosphorylation even in the absence of ligand, consistent with previous reports^48^,^49^,^51^. The phosphorylation increased by a factor of ∼1.5 to ∼3 on treatment with fgf1, and by a factor of ∼2 to ∼4 on treatment with fgf2.

Unlike the cases of FGFR1 and FGFR3, the phosphorylation of FGFR2 was the same whether the receptor was unliganded or was liganded by either fgf1 or fgf2 (*P*>0.1; Supplementary Fig. 6). This finding is consistent with the literature^45^, and may be due to the fact that FGFR2 interacts with soluble adaptor proteins, such as Grb2, which can regulate its dimerization and activity^44^,^45^. These adapter proteins are present in the activation/western blot experiments that probe the overall biological response of the receptors to their ligands, but they are absent from the plasma membrane-derived vesicles, which do not contain cell cytoplasm^34^.

### Ligand binding triggers a switch in the FGFR3 TM dimer

The only FGFR TM domain high-resolution dimer structure reported thus far is the one for the TM domain of FGFR3 (ref. 46). In this structure, solved by NMR in micelles, the FGFR3 TM helices form a left-handed dimer, with helix–helix interactions occurring along the entire TM domain. The TM helices are almost parallel, and wrap around each other in a tight, closed-packed configuration (Supplementary Fig. 7). Importantly, the TM domain of FGFR3 also contains several GxxxG-like motifs, sometimes called SMALLxxxSMALL motifs. They have small amino acids such as Gly, Ala, Thr and Ser in *i*, *i*+4 positions, and are capable of driving interactions between hydrophobic helices in membranes^52^,^53^,^54^,^55^,^56^. The FGFR3 TM domain has four such motifs in the N-terminal part of the TM helix (shown in colour in Supplementary Fig. 7C). While they do not participate in the NMR interface, they have been proposed to form an alternative dimer interface for FGFR3 (ref. 46).

**Figure 4:**
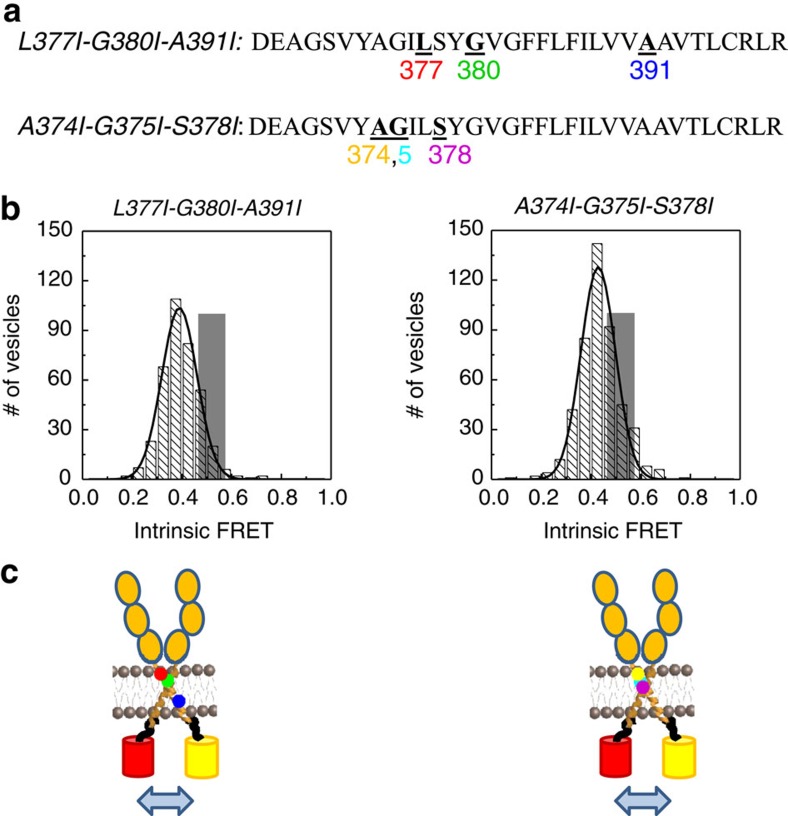
The L377I-G380I-A391I and the A374I-G375I-S378I sets of mutations affect the unliganded dimer state. (**a**) Sequence of FGFR3 TM domain, with the mutations that were engineered in this study underlined. The L377I-G380I-A391I set of mutations (left) were engineered to destabilize the interface in the FGFR3 dimer structure, solved for the isolated TM domain in detergent micelles^46^. The A374I-G375I-S378I mutations (right) were engineered to destabilize a putative alternative dimer structure, mediated by GxxxG-like motifs^46^. (**b**) Intrinsic FRET values measured for the L377I-G380I-A391I (left) and A374I-G375I-S378I (right) mutants in the absence of ligand. Dark grey Gaussians: histograms of single-vesicle intrinsic FRET measured for the constitutively dimeric EC+TM L377I-G380I-A391I (left) and A374I-G375I-S378I (right) mutants in the absence of ligand (Supplementary Figs 8 and 9). Grey bars: intrinsic FRET for the wild-type EC+TM, obtained by fitting the FRET data to a dimerization model (Table 1). The width of the bar represents the standard error from the fit. The intrinsic FRET decreases due to both mutations, suggesting that the fluorescent proteins in the mutant dimers move away from each other due to the mutations. (**c**) Graphic representation of the effect of the mutations on structure, indicating an increase in separation between the fluorescent proteins. Cartoons are not drawn to scale.

**Figure 5:**
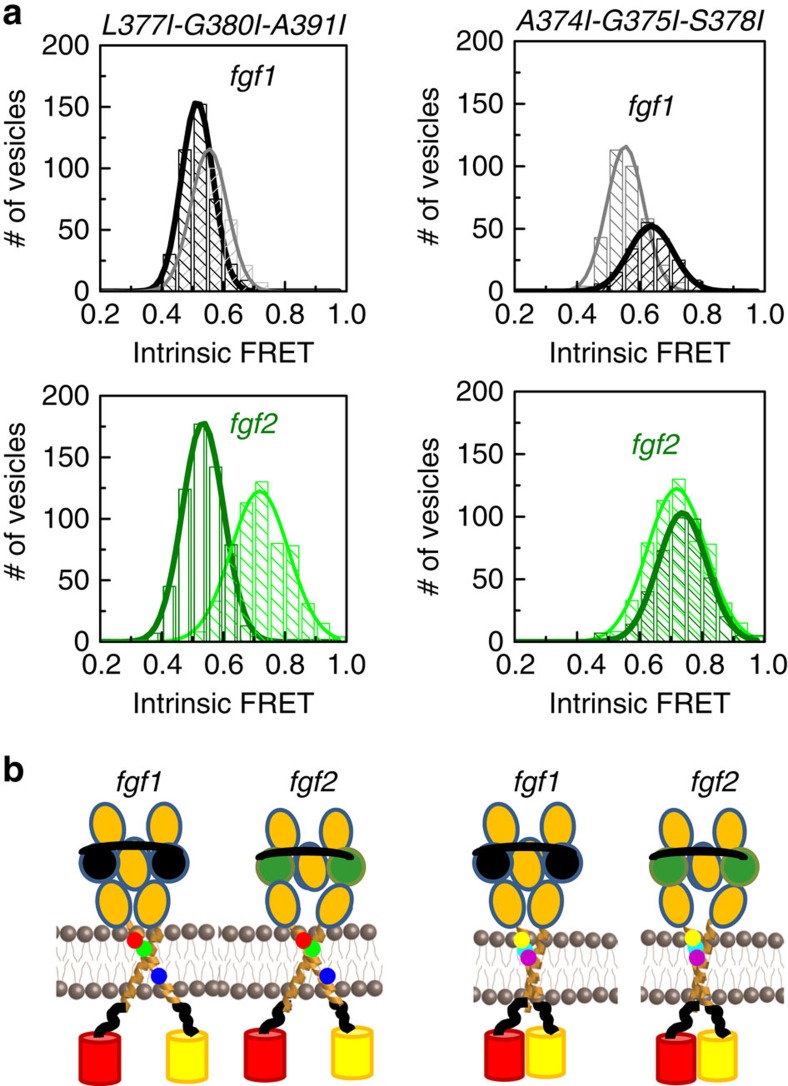
Effect of the L377I-G380I-A391I and A374I-G375I-S378I mutations on the fgf1- and fgf2-bound FGFR3 dimer structures. Left: results for the L377I-G380I-A391I mutant. Right: results for the A374I-G375I-S378I mutant. (**a**) Intrinsic FRET values, measured for the truncated EC+TM FGFR3 mutants. The histograms of measured intrinsic FRET values in single vesicles for the wild type are shown in grey for the fgf1 case and in green for the fgf2 case. The histograms for the mutants are shown in black in the presence of fgf1 and in olive in the presence of fgf2. The L377I-G380I-A391I set of mutations (left) decreases the intrinsic FRET in the presence of fgf2, down to fgf1 wild-type levels. The A374I-G375I-S378I set of mutations (right) increases the intrinsic FRET in the presence of fgf1. (**b**) Graphic representation of the findings that the L377I-G380I-A391I mutations abolish the fgf2-bound state and induce a transition to the fgf1-bound state, while the A374I-G375I-S378I mutations abolish the fgf1 state. Cartoons are not drawn to scale.

To investigate if either the observed NMR interface or the putative GxxxG-like interfaces are related to the structures that we observed in our experiments, we created two sets of amino-acid mutations, each designed to destabilize one of the two TM dimer interfaces. First, we mutated residues L377, G380 and A391 to Ile. These residues mediate the interactions between the TM helices in the NMR structure^46^. Second, we mutated A374, G375 and S378 to Ile, to eliminate all GxxxG-like motifs in the N-terminal portion of the FGFR3 sequence (Supplementary Fig. 7). The comprehensive QI-FRET characterization of the dimerization of the EC+TM NMR interface L377I-G380I-A391I mutant and the GxxxG-like A374I-G375I-S378I mutant, in the absence and presence of fgf1 or fgf2, is shown in Supplementary Figs 8 and 9. Histograms of single-vesicle intrinsic FRET values for the two mutants are shown in Fig. 4 (unliganded) and Fig. 5 (liganded), and in Table 2.

In the absence of ligand, both sets of mutations significantly stabilized the EC+TM FGFR3 unliganded dimers, rendering them constitutively dimeric (Supplementary Figs 8 and 9). Both sets of mutations decreased the intrinsic FRET, indicating that the fluorescent proteins were further away from each other in the mutants, as compared with the wild type (Fig. 4). The fact that mutations in both the NMR interface, and the alternate GxxxG interface, had very significant effects on unliganded FGFR3 dimerization suggests that the dimerization interface in the unliganded EC+TM FGFR3 dimer in the plasma membrane does not map exclusively onto either one of these putative interfaces.

In the presence of saturating fgf1 concentrations, the NMR interface mutations did not affect the measured intrinsic FRET values (Fig. 5). The GxxxG-like interface mutations, however, had a significant effect on the intrinsic FRET. Thus, the TM dimer interface in the fgf1-bound dimer does not involve the amino acids in the NMR interface, but instead likely involves the alternative GxxxG-like motifs.

In the presence of saturating fgf2 concentrations, the NMR interface mutations lead to a decrease in intrinsic FRET. As a result, the intrinsic FRET for the NMR interface mutant is identical in the presence of fgf1 or fgf2, and is also identical to the intrinsic FRET value for the wild type in the presence of fgf1. On the other hand, mutating the GxxxG-like motifs had no effect on intrinsic FRET in the presence of fgf2. The mutagenesis results are consistent with the idea that the NMR interface is used by the FGFR3 dimer in the fgf2-bound state. This is supported by the highest intrinsic FRET observed in the fgf2-bound state, suggesting tight packing between the C termini of the TM helices, as in the NMR structure.

### Effect of a pathogenic mutation

The pathogenic A391E mutation in the TM domain of FGFR3 has been linked to Crouzon syndrome with acanthosis nigricans, and to bladder cancer^8^,^57^. Previous QI-FRET studies have shown that this mutation stabilizes the unliganded EC+TM FGFR3 dimer by −1.4 kcal mol^−1^ (ref. 36). The two-parameter fit of the QI-FRET data in the absence of ligand for this mutant yielded an intrinsic FRET value of 0.72±0.02, the same as the value measured here in the fgf2-state for the wild type (0.72±0.01). This fact prompted us to further investigate the behaviour of the mutant. Using QI-FRET, we characterized the intrinsic FRET of the A391E EC+TM FGFR3 construct in the presence of saturating fgf1 and fgf2 concentrations. We also characterized the phosphorylation of the full-length mutant receptor in the presence of saturating fgf1 and fgf2 concentrations.

**Figure 6:**
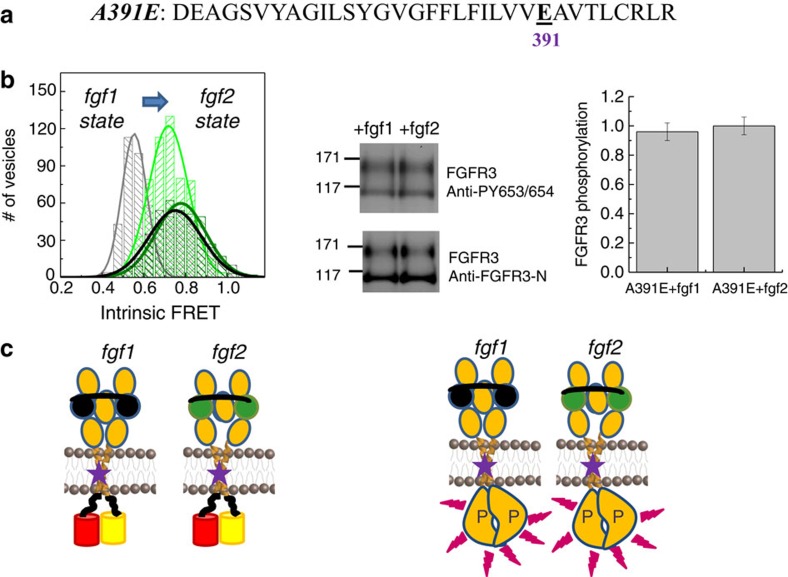
Effect of the pathogenic A391E FGFR3 mutation on dimer structures in the fgf1- and fgf2-bound states. The A391E mutation is the genetic cause for Crouzon syndrome with acanthosis nigricans, a cranial abnormality^57^, and has been linked to bladder cancer^8^. (**a**) Sequence of the A391E TM domain, with the mutation underlined. (**b**) Intrinsic FRET values, measured for the A391E EC+TM mutant in the presence of saturating concentrations of fgf1 or fgf2. The histograms for the wild type are shown in grey in the presence of fgf1 and in green in the presence of fgf2. The histograms for the A391E mutant are shown in black in the presence of fgf1 and in olive in the presence of fgf2. The intrinsic FRET values measured for the A391E mutant in the presence of fgf1 shift up, such that they overlap with the fgf2 wild-type values. Thus, the A391E mutation abolished the fgf1-bound state. (**b**) Western blots showing expression, as assayed by anti-FGFR3 antibodies, and phosphorylation of the tyrosines in the activation loop, as assayed by anti-phospho-Y653/4 antibodies. The phosphorylation of the mature fully glycosylated A391E mutant (top bands) is identical in the fgf1 and fgf2-bound states (*P*>0.01), and is the same as the phosphorylation of the wild type in the fgf2-bound state. Data are from three independent experiments. Thus, the A391E mutation increases the phosphorylation in the fgf1 state to fgf2-state levels. (**c**) Graphic representation of the finding that the A391E mutation abolishes the fgf1-bound state and induces a transition to the fgf2 state. Left: graphic representation of the finding that the average distance between the fluorescent proteins is the same in the presence of both fgf1 and fgf2. Distances are not drawn to scale. Right: graphic representation of the finding that phosphorylation is also the same in the presence of fgf1 and fgf2. The representation of the kinase domains is not based on structural data.

The comprehensive characterization of the dimerization of the A391E pathogenic mutant, in the absence and presence of fgf1 and fgf2, is shown in Supplementary Fig. 10. The intrinsic FRET values as well as the western blots that report the phosphorylation of the mutants in the presence of fgf1 or fgf2 are shown in Fig. 6. The intrinsic FRET values in the fgf1 and fgf2 bound states were identical for this mutant, and were the same as the intrinsic FRET value for wild-type EC+TM in the presence of fgf2 (Fig. 6b). The phosphorylation of the full-length A391E mutant in the presence of saturating fgf1 or fgf2 concentrations was also the same, and was the same as in the wild-type fgf2-bound state (Fig. 6b). Specifically, the phosphorylation levels of fgf1- and fgf2-bound A391E FGFR3 were 1.14±0.07 and 1.04±0.07, respectively, scaled to wild-type FGFR3 phosphorylation of 1.0. These differences were not statistically significant, *P*=0.38. Thus, the A391E mutation increased FGFR3 phosphorylation in the presence of fgf1, up to fgf2 levels.

Taken together, published data^36^ and the results reported here show that the A391E mutation abolishes the fgf1 state and traps the FGFR3 dimer in the fgf2 state even in the absence of ligand. This finding can be explained by the formation of a stabilizing hydrogen bond between the mutant Glu and the neighbouring helix, an idea that is supported by molecular modelling^58^. Once formed, this structure does not change significantly on binding fgf1 or fgf2. The A391E mutation therefore mimics the action of fgf2 in enforcing a close-packed TM dimer structure that leads to increased phosphorylation and thus disregulated signalling and disease.

### Correlation between structure and phosphorylation

**Figure 7:**
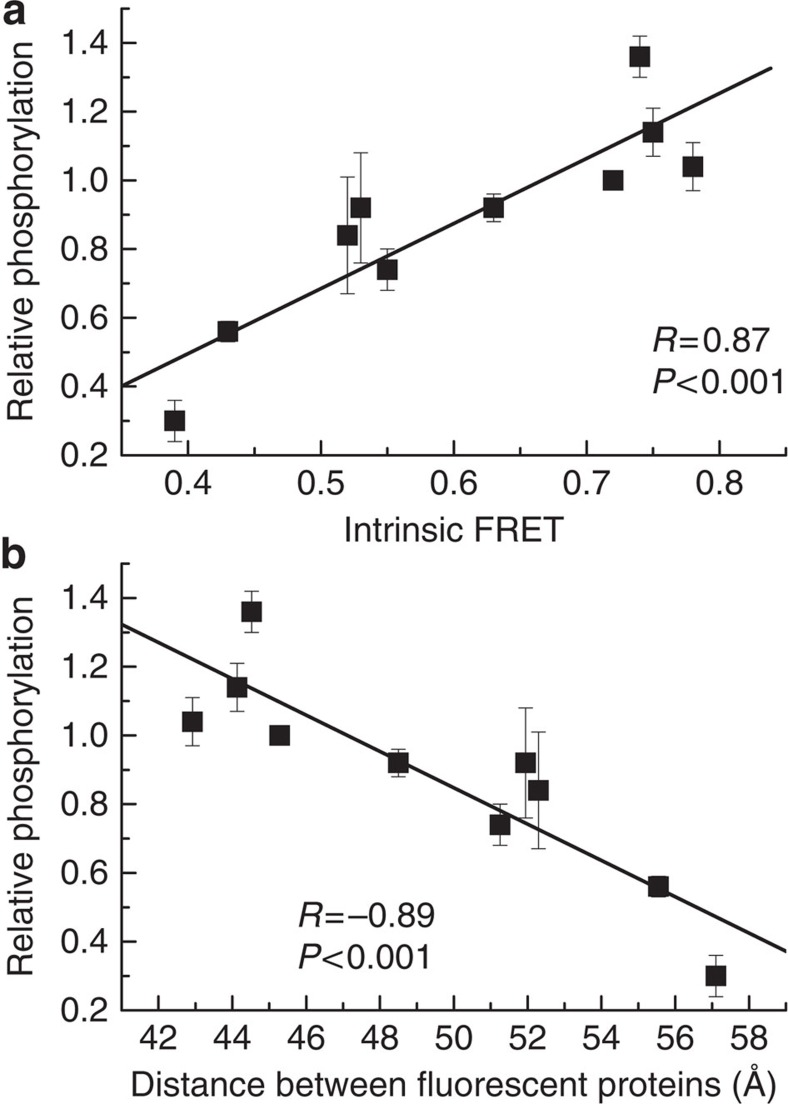
Correlation between intrinsic FRET and phosphorylation. Results are shown for wild-type FGFR3 and the three studied FGFR3 mutants: the L377I-G380I-A391I mutant, the A374I-G375I-S378I mutant, and the A391E mutant, when 100% dimeric (see text). At least three independent experiments were performed for each mutant. The phosphorylation of the wild type in the fgf2-bound state is assigned a value of 1, and all other measured phosphorylation levels are scaled accordingly. (**a**) There is a strong correlation between the measured intrinsic FRET and phosphorylation (*P*<0.001). (**b**) Strong correlation between the distance between fluorescent proteins in the EC+TM FGFR3 constructs and full-length FGFR3 phosphorylation.

Finally, we sought to determine if a global correlation exists between the measured intrinsic FRET of the various EC+TM FGFR3 constructs and the phosphorylation of the equivalent full-length FGFR3. We therefore compared the intrinsic FRET and the phosphorylation of all FGFR3 variants that exhibited constitutive dimerization. These included all fgf1- and fgf2-bound FGFR3 variants (the wild type, the pathogenic A391E mutant, the L377I-G380I-A391I mutant and the A374I-G375I-S378I mutant), as well as the constitutive unliganded dimers of the L377I-G380I-A391I and the A374I-G375I-S378I mutants. Phosphorylation, measured using western blotting, was normalized to the phosphorylation of fgf2-bound wild type, which was assigned a value of 1. An example of a western blot, used to arrive at the relationship, is shown in Supplementary Fig. 11. The final results, shown in Fig. 7a, reveal a strong correlation between intrinsic FRET and phosphorylation (*P*<0.001, *R*^2^=0.8). In Fig. 7b, we show the same correlation, but we plot the calculated distance between fluorescent proteins in the EC+TM FGFR3 dimers, instead of their intrinsic FRET.

## Discussion

Since the discovery of receptor tyrosine kinases (RTKs) in the 1970s, researchers have been searching for a model that captures the essence of RTK signal transduction across the plasma membrane. The most widely accepted model is the ‘diffusion-based' or ‘canonical' model of RTK activation^16^. It postulates that RTKs are monomers in the absence of ligand, but dimerize and cross-phosphorylate/activate each other on ligand binding. Here we demonstrate, however, that FGFR dimers exist in the absence of ligand at physiological concentrations (<100 receptors per square micron, see Fig. 1). These unliganded dimers are stabilized through contacts between the TM domains and the IC domains (Fig. 2). We also show that the unliganded FGFR dimers are phosphorylated, providing an explanation of the fact that FGFR overexpession leads to cancer^10^,^11^,^12^,^13^,^14^,^15^. The dimers undergo structural changes in response to ligand binding, and these structural changes increase phosphorylation. These observations are consistent with the ‘pre-formed dimer' model of RTK activation^44^,^59^,^60^,^61^.

The dimerization propensities of FGFR1, FGFR2 and FGFR3 in the absence of ligand are very different. Indeed, FGFR1 is predominantly monomeric at low physiological expression levels (Fig. 1). This finding suggests that the difference between the ‘canonical' and the ‘pre-formed dimer' models of RTK activation may not be fundamental, but may simply lie in the magnitude of the dimerization constant for unliganded receptors. Thus, all RTKs may follow a universal model of activation, which includes unliganded dimers of various stabilities as intermediates. The hypothesis that such a universal model can describe the activation mechanism of the different RTKs is testable with the QI-FRET method used here.

We provide a direct experimental demonstration that ligand-induced structural changes occur in FGFR dimers within the plasma membrane. Ultimately, the ligand controls the structure of the TM domain by triggering a switch to a specific configuration, and the resulting structure of the TM dimer controls the activity of the receptor. The structural changes in response to fgf1 and fgf2 binding are very different, resulting in different distances between the intracellular domains, and different phosphorylation levels for the fgf1- and fgf2-bound dimers. Thus, there exist multiple active ligand-bound states for the FGF receptors.

**Figure 8:**
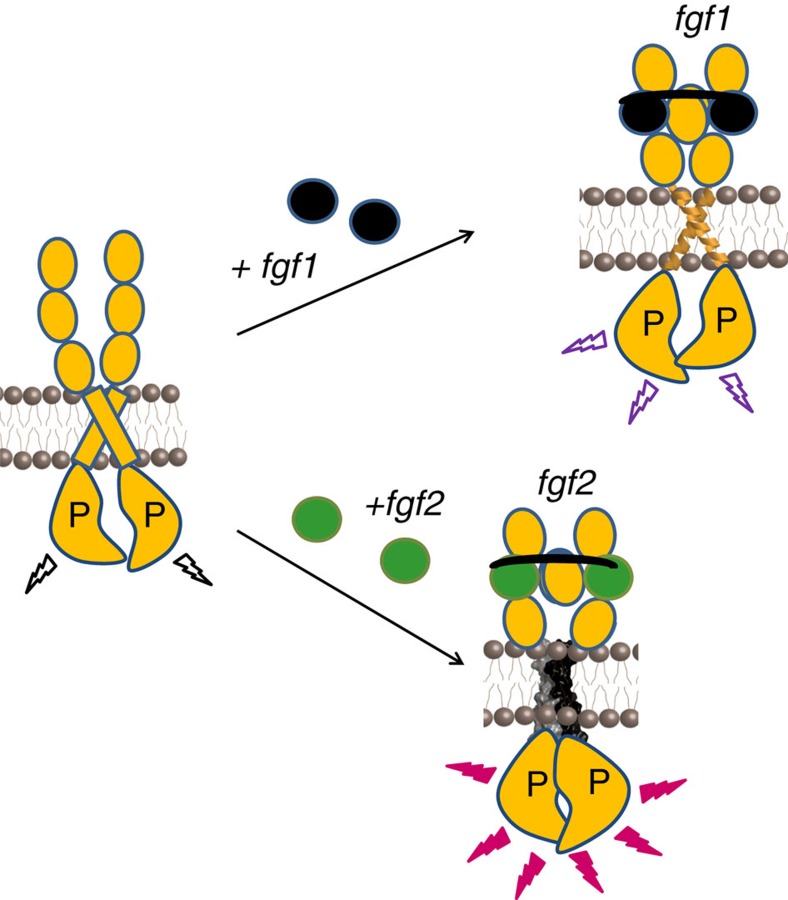
The mechanism of FGFR3 activation. Top: on fgf1 binding to extracellular domains D2 and D3, and the D2–D3 linker^26^,^69^, FGFR3 TM domains change their configuration and engage in interactions that involve small residues in the N-terminal portion of the TM domains. FGFR3 dimer phosphorylation increases by a factor of ∼1.5 to ∼3. Bottom: binding of fgf2 to D2, D3, and the linker, on the other hand, triggers a switch towards a closely packed TM dimer structure. Contacts between the TM helices are likely mediated by L377, G380 and/or A391, as in the case of a published NMR structure of the isolated TM domain^46^. FGFR3 dimer phosphorylation increases by a factor of ∼2 to ∼4.

The FRET experiments demonstrate that FGFR3 TM domains can form at least three different dimer structures, corresponding to the unliganded, fgf1- and fgf2-bound states. On fgf1 binding, FGFR3 TM domains change conformation and engage in interactions that likely involve small amino-acid residues in the N-terminal portion of the receptors (Fig. 8). FGFR3 phosphorylation increased by a factor of 1.5–3 on treatment with saturating fgf1 concentrations. We note that this cannot be explained by an increase in dimerization because FGFR3 dimerization is already very high under these conditions. Indeed, the dimeric fractions in QI-FRET experiments with full-length FGFR3 exceed 80% (Fig. 1), and phosphorylation experiments were done at similar expression levels (assessed by western blotting). We thus conclude that the structural change that occurs in the preformed FGFR3 dimer on fgf1 binding increases FGFR3 phosphorylation. Binding of fgf2 triggers a structural change towards a different TM dimer structure in which the interface likely involves contacts between L377, G380 and A391 (Fig. 8). The fgf2-bound FGFR3 dimer structure ensures the smallest separation between the TM domains and the highest FGFR3 phosphorylation, which increases by a factor of 2–4.

The difference between the fgf1- and fgf2-bound states observed here provides an explanation of the different biological roles of these two ligands. Hidai *et al.*^28^ studied the effects of fgf1 and fgf2 on the differentiation of a multipotent embryonal carcinoma cell line, and found that fgf1 promotes differentiation into cardiac muscle cells, while fgf2 induces differentiation into skeletal muscle cells. Our study suggests that these profoundly different biological effects may originate in structural differences of the receptor–ligand complexes on the cell surface.

An important result of this study is the strong correlation between intrinsic FRET and kinase phosphorylation (Fig. 7). Since in the full-length receptors the kinase domains are attached to the TM domains via the juxtamembrane domains, these results suggest that a correlation exists between the separation of the kinase domains in the dimer and their phosphorylation: the smaller the distance between the kinases, the higher their phosphorylation. However, the distance between the TM domain C termini is not the only parameter that affects phosphorylation levels. For example, fgf1 binding to FGFR3 does not change the intrinsic FRET, but increases receptor phosphorylation. There appears to be a conformational change in the TM domain on fgf1 binding that likely affects TM helix rotation, not separation, and thus cannot be captured in the FRET experiments. These findings, and published work^62^, suggest that the relative orientation of the kinases with respect to each other is another important parameter that determines phosphorylation efficiencies.

The activation mechanism discovered here sheds new light on the effect of pathogenic FGFR mutations on FGFR signalling^6^,^63^,^64^,^65^. We show that the A391E mutation in FGFR3, linked to Crouzon syndrome with acanthosis nigricans and to bladder cancer^8^,^57^, mimics the structural and functional effects of fgf2 binding. In particular, the mutation prevents the FGFR3 dimer from exploring the unliganded and fgf1-bound conformations, and traps it in its most active state, the fgf2 state. This is a fundamentally novel mechanism through which FGFR-linked pathologies can arise.

## Methods

### Plasmids

The YFP plasmid was received from Dr M. Betenbaugh (Johns Hopkins University, Baltimore, MD) and the pRSET-mCherry plasmid was obtained from Dr R.Tsien (University of California, San Diego). The plasmids encoding human wild-type FGFR1 IIIc and FGFR2 IIIc in the pRK5 vector were received from Dr M. Mohammadi, NYU. The plasmid encoding human wild-type FGFR3 IIIc in the pcDNA3.1(+) vector was a gift from Dr D. J. Donoghue, UCSD. All primers were purchased from Invitrogen.

For this work, the full length *FGFR1* and *FGFR2* genes were cloned into the pcDNA3.1(+) vector. To accomplish this, the genes were first amplified using polymerase chain reaction (PCR) and then each gene was double digested using Hind III and XhoI restriction enzymes and ligated into the pcDNA3.1(+) vector. *YFP* or *mCherry* genes were subsequently fused to the C-terminal tail of each receptor via a 3-amino-acid (GGS) linker (Supplementary Fig. 1) between the XhoI and XbaI restriction sites in the vector. The A206K mutation was introduced in the *YFP* gene to render it monomeric.

Details on the cloning of *FGFR3 EC+TM-(GGS)*_*5*_*-YFP* and *FGFR3 EC+TM-(GGS)*_*5*_*-mCherry* into the pcDNA3.1(+) vector are given elsewhere^33^. For this work, the sequences encoding for the EC and TM domains of FGFR1 and FGFR2 were amplified by PCR, double digested using HindIII and EcoRV restriction enzymes and inserted in place of the *FGFR3 EC+TM* gene in the *FGFR3 EC+TM-(GGS)*_*5*_*-YFP* and *FGFR3 EC+TM-(GGS)*_*5*_*-mCherry* plasmid constructs, to produce the *FGFR1 EC+TM-(GGS)*_*5*_*-YFP*, *FGFR1 EC+TM-(GGS)*_*5*_*-mCherry*, *FGFR2 EC+TM-(GGS)*_*5*_*-YFP* and *FGFR2 EC+TM-(GGS)*_*5*_*-mCherry* plasmids (Supplementary Table 1).

All the mutant full-length *FGFR3* gene constructs were created from the wild type using QuickChange II XL Site-Directed Mutagenesis Kit (Stratagene, CA). These constructs are shown in Supplementary Fig. 2, and are used in the western blotting experiments.

The EC+TM (A374I-G375I-S378I) FGFR3 mutant and the EC+TM (L377I-G380I-A391I) FGFR3 mutant used in the FRET studies were generated from the full-length mutants. The complementary DNA (cDNA) encoding the EC and mutant TM domains was amplified using PCR and was double digested with HindIII and EcoRV. The *FGFR2 EC+TM-(GGS)*_*5*_*-YFP* and *FGFR2 EC+TM-(GGS)*_*5*_*-mCherry* plasmid constructs were also double digested with HindIII and EcoRV enzymes, and ligated with the PCR products.

Details on the cloning of *FGFR3 TM-(GGS)*_*5*_*-YFP* and *FGFR3 TM-(GGS)*_*5*_*-mCherry* into the pcDNA3.1(+) vector are given elsewhere^40^. For this work, the sequences encoding for the TM domains of FGFR1 and FGFR2 were amplified by PCR, double digested using KpnI and EcoRV restriction enzymes and ligated with *pcDNA-(GGS)*_*5*_*-mCherry* and *pcDNA-(GGS)*_*5*_*-YFP* vectors to yield the *FGFR1 TM-(GGS)*_*5*_*-YFP*, *FGFR1 TM-(GGS)*_*5*_*-mCherry*, *FGFR2 TM-(GGS)5-YFP* and *FGFR2 TM-(GGS)*_*5*_*-mCherr*y plasmids.

### Cell culture and transfection for FRET experiments

CHO cells were cultures at 37 °C with 5% CO_2_ for 24 h. Transfection was carried out using Fugene HD transfection reagent (Roche Applied Science), following the manufacturer's protocol. Cells were co-transfected with 3–7 μg of DNA encoding receptors tagged with either YFP or mCherry.

### Production of mammalian plasma membrane vesicles

Vesiculation was performed using a chloride salt vesiculation buffer consisting of 200 mM NaCl, 5 mM KCl, 0.5 mM MgSO_4_, 0.75 mM CaCl_2_, 100 mM bicine and protease inhibitor cocktail (Complete mini EDTA-free tabs, Roche Applied Science) adjusted to pH of 8.5 (ref. 38). CHO cells were rinsed twice with 30% PBS (pH 7.4), and incubated with 1 ml of chloride salt vesiculation buffer overnight at 37 °C. A large number of vesicles were produced after 12 h, and the vesicles were transferred into four-well Nunc Lab-Tek II chambered coverslips for imaging. Images of vesicles with FGF receptors tagged with fluorescent proteins are shown in Supplementary Fig. 3. The cytoplasm in the vesicles is lost during vesicle production, as attested by the fact that soluble proteins of molecular weight up to 200 kDa are not retained inside the vesicles^34^.

### QI-FRET image acquisition

Vesicles were imaged using a Nikon Eclipse confocal laser scanning microscope using a × 60 water immersion objective. All the images were collected and stored at a 512 × 512 resolution. Three different scans were performed for each vesicle: (1) excitation at 488 nm, with a 500–530-nm emission filter (donor scan); (2) excitation at 488 nm, with a 565–615-nm emission filter (FRET scan); and (3) excitation at 543 nm, with a 650-nm longpass filter (acceptor scan). Gains of 8.0 were used for all the three scans. The bleaching of the fluorescent proteins was minimized through the use of ND8 filters when exciting with the 488-nm laser, and low pixel dwell time (1.68 μs).

### QI-FRET data analysis

The QI-FRET method has been described previously^33^,^41^, but was applied here for the first time to full-length FGFRs. Purified solutions of YFP and mCherry^66^of known concentration were imaged in the donor, FRET and acceptor scans, to obtain the calibration constants for the donor and the acceptor, *i*_D_ and *i*_A_, as well the bleed-through coefficients for the donor and the acceptor, *β*_D_ and *β*_A_ (ref. 37). A soluble linked YFP–mCherry protein was also imaged in the three channels to obtain the gauge factor *G*_F_ as described by Li *et al.*^37^

Each vesicle co-expressing FGFR–YFP and FGFR–mCherry was imaged in the donor, acceptor and FRET channels (Supplementary Fig. 3). The fluorescence intensities across the plasma membrane, *I*_D_, *I*_FRET_ and *I*_A_, in the three channels, were determined as described in detail elsewhere^33^. The acceptor concentration in each vesicle, *C*_A_, was calculated according to ref. 33:


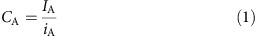


The sensitized emission of the acceptor in each vesicle was determined as^37^:


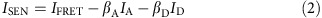


The donor intensity in the absence of the acceptor *I*_D,corr_, and the donor concentrations (*C*_D_) were calculated as:


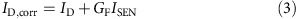



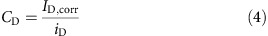


From equations (1) and (4), the total concentration, *T*, and the acceptor fraction, *x*_A_, are calculated according to:


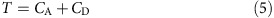



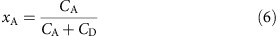


The FRET efficiency, *E*, was calculated using equation (7):


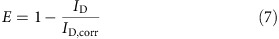


The FRET efficiency was corrected for the so-called ‘proximity FRET' contribution, which describes the close approach of donors and acceptors (within distances of 100 Å or so) in the membrane without specific interactions^67^. The dimeric fraction is determined from the corrected FRET efficiency according to:


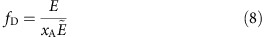


The constant 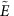 in equation (8) is the ‘intrinsic FRET', the FRET efficiency in a dimer containing a donor and an acceptor. This is a structural parameter, a constant for each receptor dimer, which depends only on the separation and the orientation of the two fluorescent proteins in the dimer, not on the dimerization propensity. *x*_A_ is the acceptor fraction.

Based on the law of mass action, the dimeric fraction can be written as a function of the total receptor concentration, *T*, and the dimerization constant *K* according to equation (9):


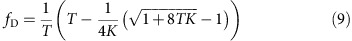


Equations (8) and (9) are used to fit the dimerization model to the data while optimizing for two adjustable parameters: the dimerization constant *K*, and the intrinsic FRET, 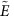 (Supplementary Fig. 4). The dissociation constant *K*_diss_*=1/K* is reported in units of receptors per μm^2^ in Table 1. The value of *K*_diss_ can be then directly compared with expressions levels in order to evaluate the biological significance of dimerization.

The free energy of dimerization (dimer stability) *ΔG* is calculated from the dimerization constant *K*=1/*K*_diss_. The standard state is defined as nm^2^ per receptor^33^, and therefore:


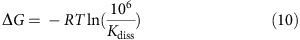


In the case of 100% dimers (*f*_D_=1), equation (8) can be re-written as:


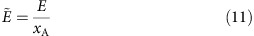


Thus, measurements of *E* and *x*_A_ for each vesicle in this case allows us to directly determine the value of the intrinsic FRET, 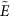, in each vesicle. Histograms of the measured 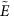 are shown throughout the manuscript, such as in Fig. 3, for example.

Finally, the dependence of the intrinsic FRET, 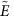, on the distance between the fluorescent proteins in the dimer is given by equation (12).


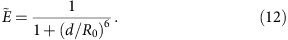


Here *d* is the distance between the acceptor and the donor in the dimer, and *R*_o_ is the Förster radius of the FRET pair^33^. For eYFP and mCherry, *R*_o_ is 53.1 Å.

### Western blots

CHO and HEK 293T cells were starved in serum-free medium for 24 h following transfection with 0.3 to 2.5 μg of DNA encoding the full-length receptors and then treated with lysis buffer (25 mM Tris-HCl, 0.5% Triton X-100, 20 mM NaCl, 2 mM EDTA, phosphatase inhibitor and protease inhibitor, Roche Applied Science). Lysates were collected following centrifugation at 15,000*g* for 15 min at 4 °C and loaded onto 3–8% NuPAGE Novex Tris-Acetatemini gels (Invitrogen, CA). The proteins were transferred onto a nitrocellulose membrane, and blocked using 5% milk in Tris-buffered saline. The phosphorylation of the tyrosines in the activation loop of the FGFR kinases was assessed first following protein transfer to the nitrocellulose membrane. Immunostaining was performed using specific anti-phospho-Tyr antibodies (Tyr653/654; #3471; Cell Signaling Technologies). These antibodies are raised against Tyr653/654 in the activation loop of FGFR1, but are reactive to all FGF receptors because of their identical activation loop sequences. Anti-phospho-Tyr766 (1E5; #2544; Cell Signaling Technology) was used to asses FGFR1 phosphorylation at Tyr766. Anti-phospho-Tyr724 (sc-33041; Santa Cruz Biotechnology) was used to detect the phosphorylation of Tyr724 in FGFR3. The membranes were then stripped and total FGFR protein levels were assessed using antibodies against FGFR3 (H-100; sc-9007), FGFR2 (H-80; sc-20735) and FGFR1 (H-76; sc-7945) from Santa Cruz Biotechnology. All the primary antibodies were followed by anti-rabbit HRP conjugated antibody (W4011, Promega). Dilutions were 1:1,000 for all primary antibodies and 1:2,500 for the secondary antibody. The proteins were detected using the Amersham ECL detection system (GE Healthcare). Uncropped versions of gels in Fig. 3b are shown in Supplementary Fig. 6.

Experimental protocols ensured that measurements were preformed in the ‘linear regime', as described in detail in the Supplementary Data file in ref. 68.

### Activation with fgf1 and fgf2

CHO and HEK 293T cells were cultured in normal medium for 24 h following transfection and then starved in serum-free medium for 24 h. Human fgf1 and human fgf2 were purchased from Cell Signaling Technologies (catalogue # 5234LC and # 8910LC, respectively). 5000, ng ml^−1^ of fgf1 or fgf2 were added to the serum-free medium. After incubating for 10 min at 37 °C with ligand, cells were placed on ice immediately and lysed as described above and analysed using western blotting.

## Additional information

**How to cite this article:** Sarabipour, S and Hristova, K. Mechanism of FGF receptor dimerization and activation. *Nat. Commun.* 7:10262 doi: 10.1038/ncomms10262 (2016).

## Supplementary Material

Supplementary Supplementary Figures 1-11, Supplementary Table 1 and Supplementary References

## Figures and Tables

**Table 1:** Parameters describing FGFR unliganded dimerization.

	***K***_**diss**_ **(rec μm**^−2^**)**	***ΔG*** **(kcal mol**^−1^**)**	**Intrinsic FRET**	**d (Å)**
Full FGFR1	710 (630 to 826)	−4.3 (−4.2 to −4.4)	0.66 (0.65 to 0.68)	47.6 (46.8 to 47.9)
Full FGFR2	111 (100 to 146)	−5.4 (−5.3 to −5.5)	0.43 (0.42 to 0.44)	55.7 (55.3 to 56.1)
Full FGFR3	24 (14 to 34)	−6.3 (−6.1 to −6.5)	0.55 (0.54 to 0.56)	51.4 (51.0 to 51.7)
ECTM FGFR1	428 (370 to 540)	−4.6 (−4.5 to −4.7)	0.5 (0.47 to 0.51)	53.1 (52.8 to 54.2)
ECTM FGFR2	3,235 (2,726 to 3,809)	−3.4 (−3.3 to −3.5)	0.57 (0.55 to 0.61)	50.7 (49.3 to 51.4)
ECTM FGFR3	3,235 (2,670 to 3,660)	−3.4 (−3.3 to −3.5)	0.52 (0.49 to 0.55)	52.4 (51.4 to 53.5)
TM FGFR1	40 (27 to 55)	−6.0 (−5.8 to −6.2)	0.5 (0.47 to 0.51)	53.1 (52.8 to 54.2)
TM FGFR2	67 (51 to 86)	−5.7 (−5.6 to −5.8)	0.52 (0.51 to 0.53)	52.4 (52.1 to 52.8)
TM FGFR3	156 (124 to 180)	−5.2 (−5.1 to −5.3)	0.65 (0.64 to 0.66)	47.9 (47.6 to 48.3)
ECTM FGFR3 A391E*	290 (251 to 360)	−4.8 (−4.7 to −4.9)	0.72 (0.7 to 0.73)	45.5 (45.0 to 46.1)

The parameters were obtained from least-square fits of a dimer model to the FRET data. *K*_diss_: two-dimensional dissociation constants*, ΔG:* dimerization free energies (dimer stabilities) calculated using equation (10), *d*: effective distance between fluorescent proteins in a dimer, calculated from the intrinsic FRET efficiencies using equation (12). Best fits are shown, along with the 67% confidence intervals (standard errors). The values shown in parenthesis are the lower and upper bounds for the confidence intervals, determined from the fit.

^*^Data from ref. 36.

**Table 2:** Intrinsic FRET efficiencies in the presence of saturating fgf1 or fgf2 concentrations.

	**Intrinsic FRET**	***d*** **(Å)**
ECTM FGFR1+fgf1	0.55±0.01	51.4±0.4
ECTM FGFR1+fgf2	0.73±0.01	45.0±0.4
ECTM FGFR2+fgf1	0.53±0.01	52.0±0.4
ECTM FGFR2+fgf2	0.75±0.01	44.2±0.4
ECTM FGFR3+fgf1	0.55±0.01	51.4±0.4
ECTM FGFR3+fgf2	0.72±0.01	45.4±0.4
377I-380I-391I	0.39±0.01	57.2±0.4
377I-380I-391I+fgf1	0.52±0.01	52.4±0.4
377I-380I-391I+fgf2	0.53±0.01	52.0±0.4
374I-375I-378I	0.43±0.01	55.7±0.4
374I-375I-378I+fgf1	0.63±0.01	48.6±0.4
374I-375I-378I+fgf2	0.74±0.01	44.6±0.4
A391E+fgf1	0.75±0.01	44.2±0.4
A391E+fgf2	0.78±0.01	43.0±0.4

The intrinsic FRET efficiencies, 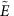, were measured for the truncated EC+TM FGFR constructs studies here. In the fgf1-bound state, the measured distance between the fluorescent proteins, d, is 51–52 Å. In the fgf2-bound state, the measured distance between the fluorescent proteins, d, is 43–45 Å. Averages are shown together with standard errors.
