# Supplementary material for: Mechanism of FGF receptor dimerization and activation
Source: Nat Commun. 2016 Jan 4;7:10262. doi: 10.1038/ncomms10262 (PMC4725768; doi:10.1038/ncomms10262)
Supplement: Supplementary — Figures 1-11, Supplementary Table 1 and Supplementary References [file ncomms10262-s1.pdf]

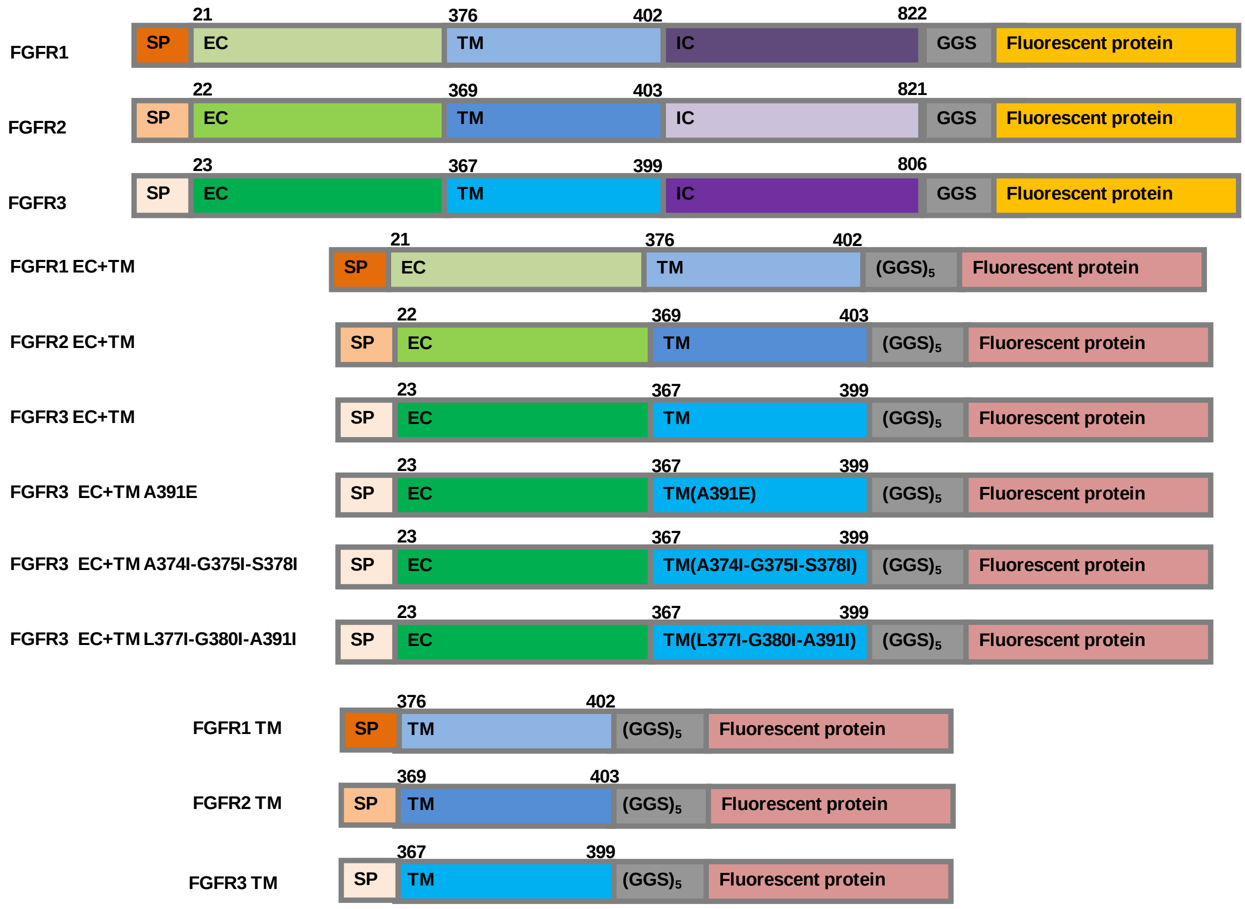

Supplementary Figure 1. The plasmid constructs used in the FRET experiments. SP: signal peptide, EC: extracellular domain, TM: transmembrane domain, IC: intracellular domain, FP: Fluorescent protein, either YFP or mCherry (a FRET pair). The full-length receptors had fluorescent proteins attached to their C-termini via a flexible GGS linker. The truncated receptors had the intracellular domain substituted with a fluorescent protein, which was attached to the TM domain via a longer flexible $(GGS)_5$ linker.

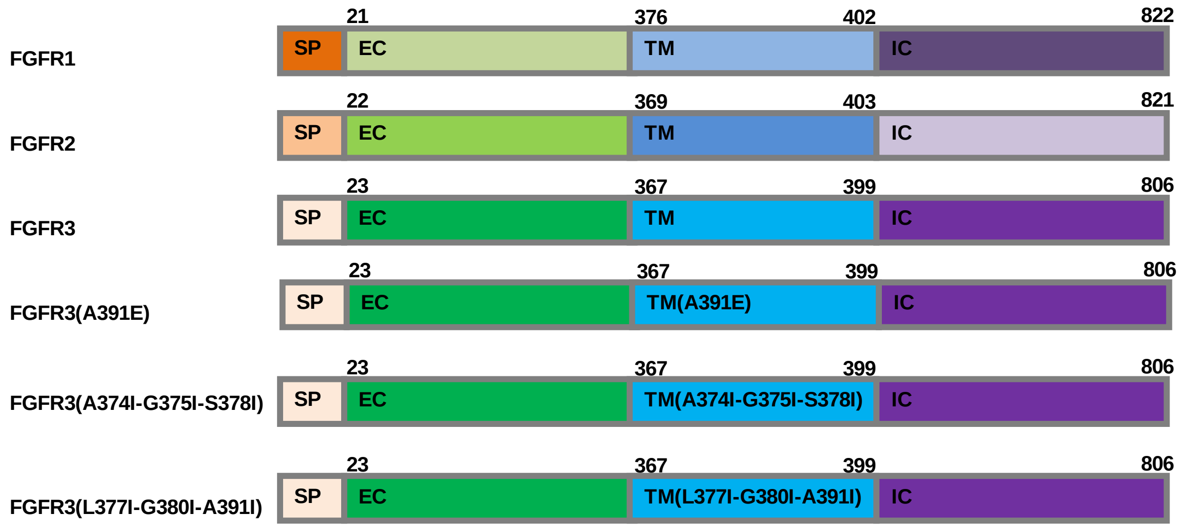

Supplementary Figure 2. The plasmid constructs used in the Western blot experiments.

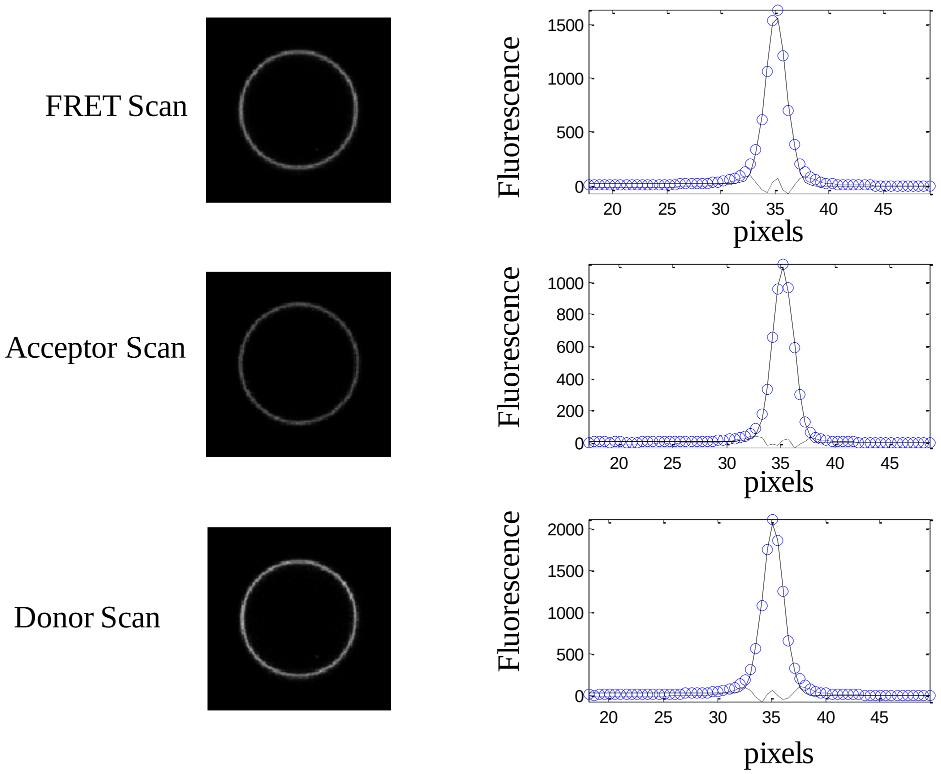

Supplementary Figure 3. **One vesicle imaged in three channels.** The vesicle co-expresses EC+TM FGFR2-YFP and EC+TM FGFR2-mCherry, and is imaged in the FRET, acceptor, and donor channels. A Gaussian function (black line) was fitted to the fluorescence intensity across the membrane (blue symbols) after correcting for background fluorescence. The residuals from the fits are shown with the dashed lines.

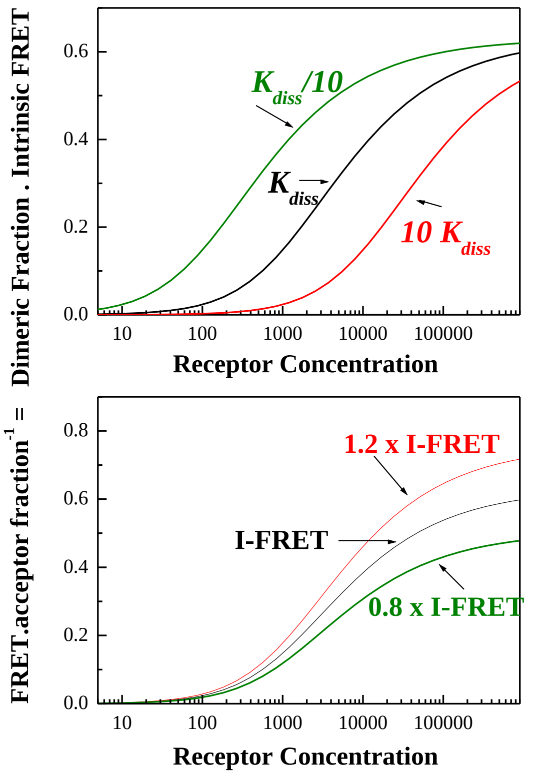

Supplementary Figure 4. **The effect of K_{diss} and Intrinsic FRET.** A visual demonstration of how the two unknown parameters, the dissociation constant K_{diss} and the Intrinsic FRET $\tilde{E}$ affect the measured FRET.acceptor fraction^{-1}.

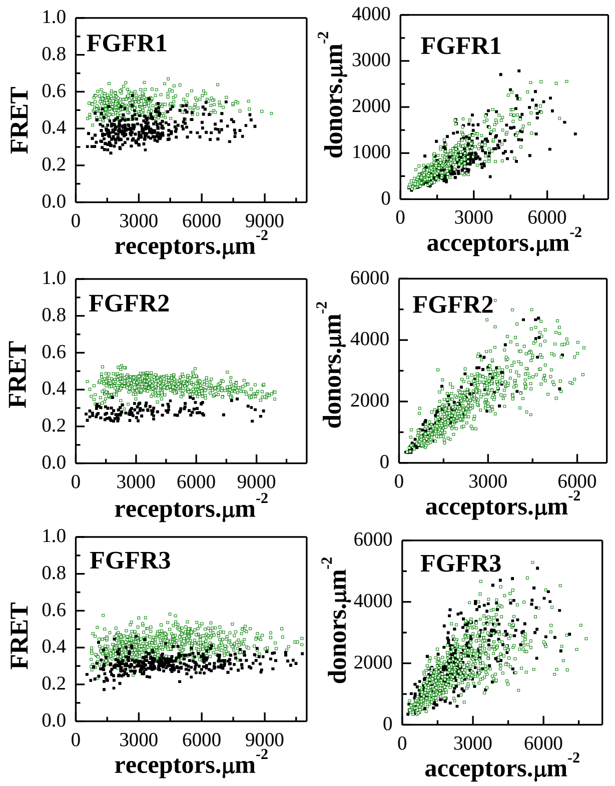

Supplementary Figure 5. **Effect of ligands on QI-FRET parameters.** FRET efficiencies, donor concentrations and acceptor concentrations, are measured for the wild-type EC+TM FGF receptors, in the presence of 5 μg.ml^{-1} fgf1 (black) or fgf2 (olive). Each data point represents a single vesicle. For each receptor/ligand pair, 300 to 500 individual plasma membrane-derived vesicles were imaged one hour after adding the ligand. FRET does not depend on the receptor concentration, as expected in the case of saturating ligand concentrations. The Intrinsic FRET for each vesicle was determined according to equation (11).

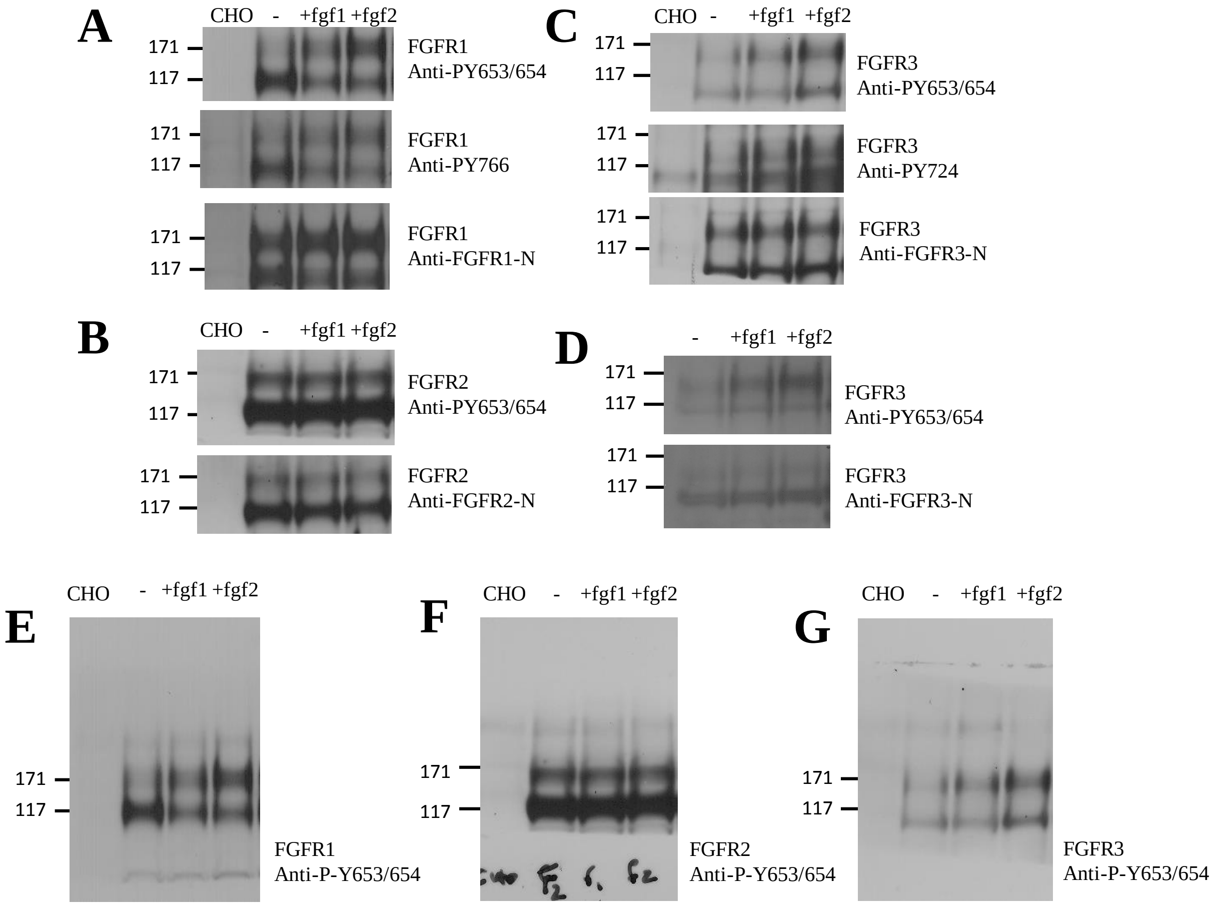

Supplementary Figure 6. FGFR phosphorylation. Phosphorylation of FGFR1, FGFR2, and FGFR3, characterized via Western blotting. Lane "CHO": no transfection; Lane "-": no ligand; Lane "fgf1": +5 $\mu g.ml^{-1}$ fgf1; Lane "fgf2": +5 $\mu g.ml^{-1}$ fgf2. (A): CHO cells transfected with wild-type FGFR1. (B): CHO cells transfected with wild-type FGFR2. (C): CHO cells transfected with wild-type FGFR3. (D): HEK 293T cells transfected with wild-type FGFR3. (E) Uncropped gel from A, top and Figure 3B, left. (F) Uncropped gel from B, top and Figure 3B, center. (G) Uncropped gel from C, top and Figure 3B, right.

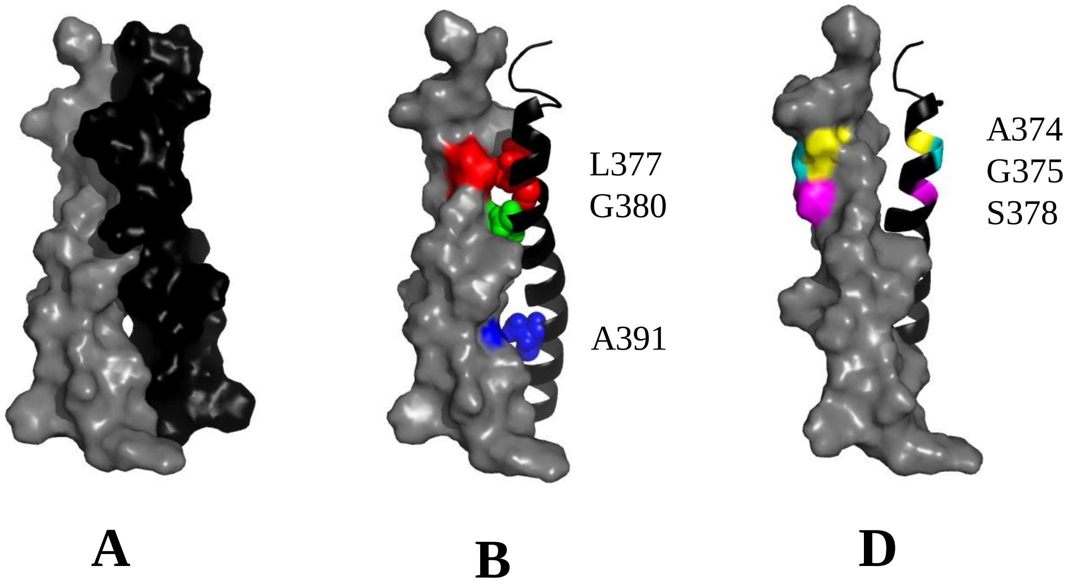

374,5

C: DEAGSVYAGILSYGVGFFLFILVVAAVTLCRLR

378

Supplementary Figure 7. **FGFR3 TM domain structure in micelles**. (A) The dimer structure, solved by NMR [1], is tightly packed. (B) Amino acids L377, G380, and A391 mediate helix-helix contacts in the structure, based on inter-helical NOEs that were measured experimentally. We mutated these three amino acids to Ile in order to disrupt this "NMR interface". (C) Amino acids A374, G375 and S378 participate in several GxxxG-like motifs (also known as SMALLxxxSMALL motifs), known to mediate TM helix dimerization [1]. These GxxxG motifs have been proposed to stabilize a putative alternative FGFR3 TM dimer structure [1]. A374, G375 and S378 were mutated to Ile in our studies in order to disrupt this putative alternative dimer interface. (D) Amino acids A374, G375 and S378 face away from the dimer interface in the NMR structure.

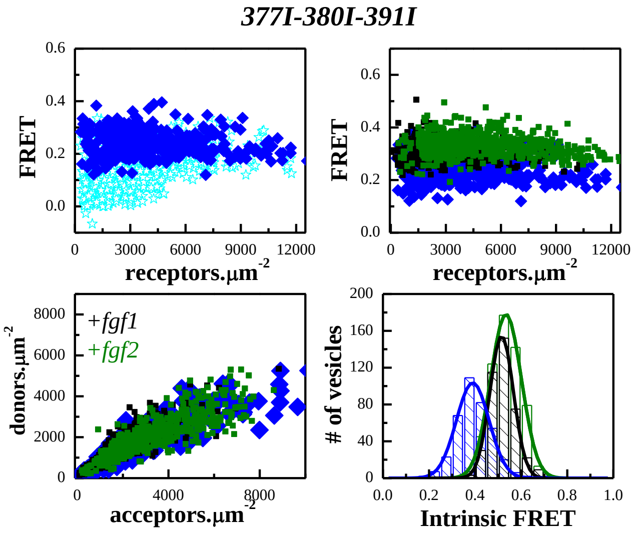

Supplementary Figure 8. **FRET data for the EC+TM FGFR3 L377I-G380I-A391I mutant.** Cyan: wild-type, no ligand. Blue: mutant, no ligand. Black: mutant + fgf1. Olive: mutant + fgf2.

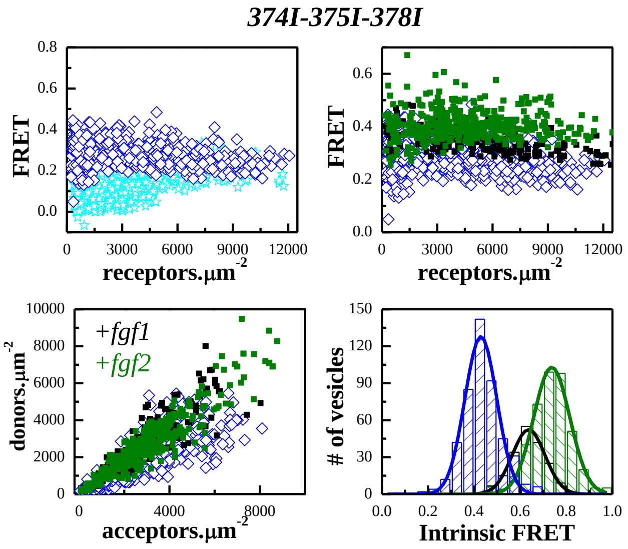

Supplementary Figure 9. **FRET data for the EC+TM FGFR3 A374I-G375I-S378I mutant.** Cyan: wild-type, no ligand. Blue: mutant, no ligand. Black: mutant + fgf1. Olive: mutant + fgf2.

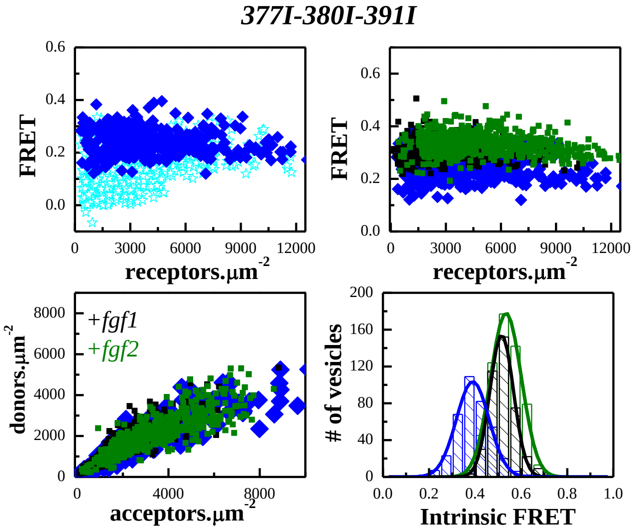

Supplementary Figure 10. FRET data for the EC+TM A391E FGFR3 mutant. The A391E mutation is the genetic cause for Crouzon syndrome with acanthosis nigricans. Magenta: wild-type, no ligand. Blue: mutant, no ligand. Black: mutant + fgf1. Olive: mutant + fgf2. Data in the absence of ligand are from [2].

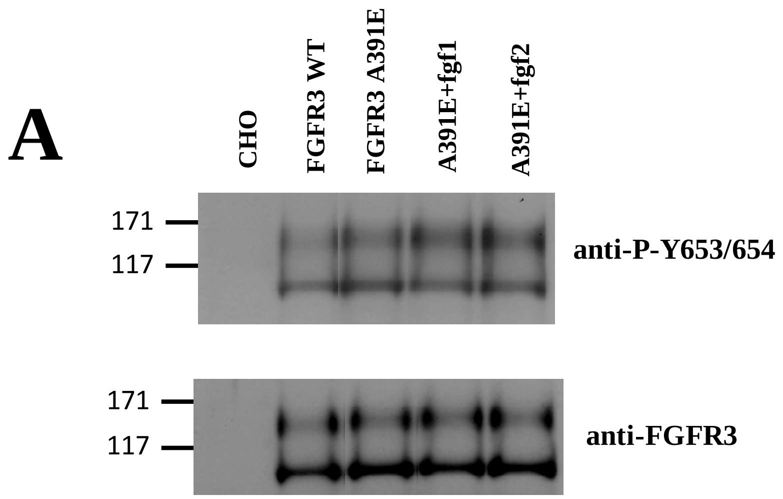

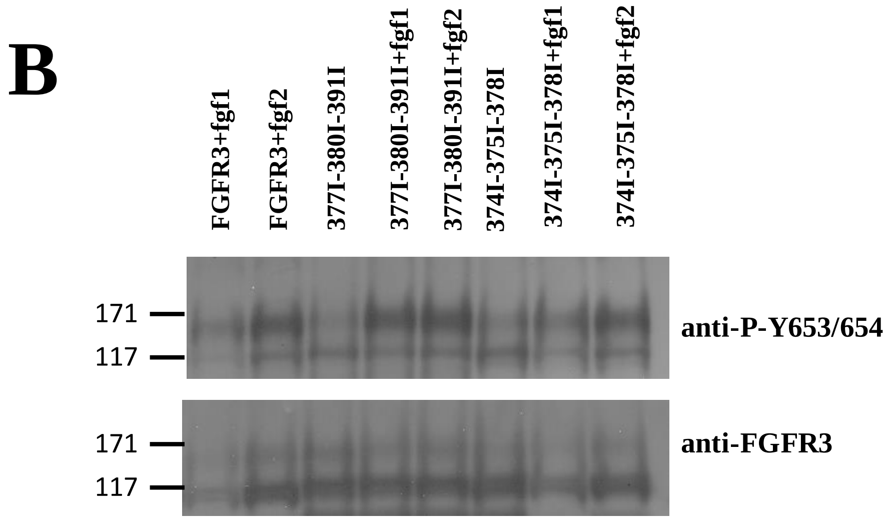

Supplementary Figure 11. **Supplementary Western blots.** (A) Western blot results for the A391E mutant. (B) Example Western blot, used to obtain the correlation in Figure 7. Phosphorylation was quantified as the ratio of the anti-phospho band intensity and the anti-FGFR3 band intensity. Only the top bands, corresponding to mature fully glycosylated FGFR3, were quantified.

Primer name	5’- 3’ sequence
FGFR1 FW	GCC AAG CTT GCC GCC ATG TGG AGC TGG AAG TGC CTC
FGFR1 RV	GCC CTC GAG GCG GCG TTT GAG TCC GCC
FGFR2 FW	GCC AAG CTT GCC GCC ATG GTC AGC TGG GGT CGT TTC
FGFR2 RV	GCC GCC CTC GAG TGT TTT AAC ACT GCC GTT TAT GTG TGG
FGFR3 FW	GCC AAG CTT GCC ACC ATG GGC GCC CCT GCC
FGFR3 RV	GGC GGC GAT ATC CGT CCG CGA GCC CCC
-(GGS)-FP FW	GCC CTC GAG GGT GGT TCA GTG AGC AAG GGC GAG GAG GAT
-(GGS)-FP RV	GGC GGC GGC TCT AGA TTA CTT GTA CAG CTC GTC CAT GCC
EC+TM FGFR1 FW	GCC AAG CTT GCC GCC ATG TGG AGC TGG AAG TGC CTC
EC+TM FGFR1 RV	GGC GGC GGC GAA TTC ACC ACT CTT CAT CTT GTA GAC GAT GAC
EC+TM FGFR2 FW	GCC AAG CTT GCC GCC ATG GTC AGC TGG GGT CGT TTC
EC+TM FGFR2 RV	GGC GGC GGC GAA TTC CGT GTT CTT CAT TCG GCA CAG GAT GAC
EC+TM FGFR3 FW	GCC AAG CTT GCC ACC ATG GGC GCC CCT GCC
EC+TM FGFR3 RV	GGC GGC GAA TTC GCG CAG GCG GCA GAG
TM FGFR1 FW	GCC GCC GGT ACC GTT GAC CGT TCT GGA AGC CCT GGA AGA GAG
TM FGFR1 RV	GGC GGC GGC GAA TTC ACC ACT CTT CAT CTT GTA GAC GAT GAC
TM FGFR2 FW	GGC GCC GGT ACC CCT GGA AGA GAA AAG GAG ATT ACA GCT TC
TM FGFR2 RV	GGC GGC GGC GAA TTC CGT GTT CTT CAT TCG GCA CAG GAT GAC
TM FGFR3 FW	GCC CGT ACG GAC GAG GCG GGC AGT GTG
TM FGFR3 RV	GGC GGC GAA TTC GCG CAG GCG GCA GAG
-(GGS)$_5$ oligonucleotide FW	GAA TTC G GGA GGA AGT GGC GGA AGT GGC GGA AGT GGC GGA AGT GGA GGA AGT GG ACC GGT
-(GGS)$_5$ oligonucleotide RV	ACC GGT CC ACT TCC TCC ACT TCC GCC ACT TCC GCC ACT TCC GCC ACT TCC TCC C GAA TTC
A391E mutagenesis FW	CTG TTC ATC CTG GTG GTG GAA GCT GTG ACG CTC TGC CGC CTG
A391E mutagenesis RV	CAG GCG GCA GAG CGT CAC AGC TTC CAC CAC CAG GAT GAA CAG
A374I mutagenesis FW	GAG GCG GGC AGT GTG TAT ATA GGC ATC CTC AGC TAC GGG
A374I mutagenesis RV	CCC GTA GCT GAG GAT GCC TAT ATA CAC ACT GCC CGC CTC

G375I mutagenesis FW	GCG GGC AGT GTG TAT GCA ATC ATC CTC AGC TAC GGG
G375I mutagenesis RV	CCC GTA GCT GAG GAT GAT TGC ATA CAC ACT GCC CGC
S378I mutagenesis FW	G TAT GCA GGC ATC CTC ATC TAC GGG GTG GGC TTC
S378I mutagenesis RV	GAA GCC CAC CCC GTA GAT GAG GAT GCC TGC ATA C
L377I mutagenesis FW	GTG TAT GCA GGC ATC ATC AGC TAC ATC GTG GGC
L377I mutagenesis RV	GCC CAC GAT GTA GCT GAT GAT GCC TGC ATA CAC
G380I mutagenesis FW	C GGC ATC CTC AGC TAC ATC GTG GGC TTC TTC CTG TTC ATC CTG
G380I mutagenesis RV	CAG GAT GAA CAG GAA GAA GCC CAC GAT GTA GCT GAG GAT GCC G
A391I mutagenesis FW	CTG TTC ATC CTG GTG GTG ATC GCT GTG ACG CTC TGC CGC CTG
A391I mutagenesis RV	CAG GCG GCA GAG CGT CAC AGC GAT CAC CAC CAG GAT GAA CAG
A206K mutagenesis in YFP FW	CTG AGC TAC CAG TCC AAA CTG AGC AAA GAC CCC
A206K mutagenesis in YFP RV	GGG GTC TTT GCT CAG TTT GGA CTG GTA GCT CAG

Supplementary Table 1. Primers used in the study.

Supplementary References

1. Bocharov,E.V. *et al.* Structure of FGFR3 Transmembrane Domain Dimer: Implications for Signaling and Human Pathologies. *Structure* **21**, 2087-2093 (2013).

2. Sarabipour,S. & Hristova,K. FGFR3 Unliganded Dimer Stabilization by the Juxtamembrane Domain. *J. Mol. Biol.* **427**, 1705-1714 (2015).
